# Two to Tango: Dialogue between Adaptive and Innate Immunity in Type 1 Diabetes

**DOI:** 10.1155/2020/4106518

**Published:** 2020-07-30

**Authors:** Lin Sun, Shugang Xi, Guangyu He, Zhuo Li, Xiaokun Gang, Chenglin Sun, Weiying Guo, Guixia Wang

**Affiliations:** Department of Endocrinology and Metabolism, The First Hospital of Jilin University, Changchun, 130021 Jilin, China

## Abstract

Type 1 diabetes mellitus (T1DM) is a long-term and chronic autoimmune disorder, in which the immune system attacks the pancreatic *β*-cells. Both adaptive and innate immune systems are involved in T1DM development. Both B-cells and T-cells, including CD4^**+**^ and CD8^**+**^ T-cells, as well as other T-cell subsets, could affect onset of autoimmunity. Furthermore, cells involved in innate immunity, including the macrophages, dendritic cells, and natural killer (NK) cells, could also accelerate or decelerate T1DM development. In this review, the crosstalk and function of immune cells in the pathogenesis of T1DM, as well as the corresponding therapeutic interventions, are discussed.

## 1. Introduction

Type 1 diabetes mellitus (T1DM) is an autoimmune disease, in which the immune system attacks the *β*-cells [1]. The incidence of T1DM has dramatically increased in recent years, which could be attributed to a certain extent to environmental factors, pathogen infections, and genetic alterations [2]. However, the exact pathogenesis of T1DM is still unclear and requires effective therapeutic interventions. Several animal models mimicking the pathological features of the disease have been developed to study the pathogenesis of T1DM [3–5]. The nonobese diabetic (NOD) mice can spontaneously develop diabetes and show similar clinical symptoms of T1DM as in humans. These symptoms include hyperglycemia, polyuria, and polydipsia [3, 6]. Both animal studies and clinical data support the notion that diverse immune cells are involved in T1DM disease progression [7, 8]. Previous studies have revealed that insulin insufficiency initiates hyperglycemia, which is driven by overactivated effector T-cells, in both clinical patients and animal models [3]. In addition, anti-CD20 therapy that depletes *Β*-cells is considered effective, suggesting the involvement of B-cells in T1DM [9]. Furthermore, natural killer (NK) cells and dendritic cells (DCs) are also involved in the process of T1DM by killing the target cells as well as interacting with T-cells [10]. Although increasing evidence has revealed that immune cells play critical roles in T1DM, the clinical outcome of targeting immune cells in T1DM treatment remains elusive. In this review, the current knowledge in innate and adaptive immunity-associated etiology and pathogenesis of T1DM as well as the corresponding therapeutic approaches are summarized.

## 2. Adaptive Immunity in T1DM

Insulitis is regarded as the pathogenic hallmark of T1DM, which occurs due to an inflammatory lesion of the pancreatic islets and loss of *β*-cells [11]. In this section, the complex function of different subtypes of adaptive immune cells, including CD4^**+**^ T-cells, CD8^**+**^ T-cells, NK T-cells, and B-cells, is depicted.

Considerable evidences have reported the pivotal role of T-cells in T1DM development [12–14]. Elevated glucose levels in the circulation results in elevated production and secretion of interleukin-1*β* (IL-1*β*) from the immune cells, which is responsible for induction of cell death [15]. T-cells, by interacting with macrophages, promote immune response against *β*-cells, leading to their destruction [16]. Cytokines, including IL-1*β* and tumor necrosis factor (TNF), induce oxidative stress by triggering the production of massive amount of reactive oxygen species (ROS), leading to cell apoptosis [17]. In summary, the inflammatory responses mediated by T lymphocytes might result in the death of pancreatic *β*-cells in T1DM and might be the primary mechanism underlying this disease.

### 2.1. CD4^+^ T-Cells

CD4^**+**^ T-cells, also known as the helper T-cells, are an important lineage of T-cells participating in B-cell class switching, CD8^+^ T-cell maturation, and facilitating the activity of macrophages. CD4^**+**^ T-cell depletion lowers the incidence of T1DM in NOD mice, and even overt diabetes, suggesting its primary role in the development of T1DM [18]. To understand the pathogenesis of T1DM, an in-depth research on proteomic profiling in a group of young T1DM diabetic patients was performed, and it revealed significant inflammation in their peripheral CD4^+^ T-cells [19]. Studies on NOD model showed that CD4^**+**^ T-cells respond to a series of *β*-cell proteins, most prominently proinsulin, included in diabetes [20]. Further studies have been conducted to investigate on how insulin and proinsulin have become the targets of autoimmune T-cell response. Delong et al. [21] screened the *β*-cell peptides that could trigger CD4^**+**^ T-cell activation and found that islet amyloid polypeptide (IAPP), chromogranin A (ChgA), and its cleaved product that is covalently hybrid with the secretory insulin from the pancreatic *β*-cells form a potent immunogenic complex and trigger the activity of CD4^**+**^ T-cells. The activated CD4^**+**^ T-cells can interact and induce the activation of DCs, facilitating the maturation and activation of CD8^**+**^ T-cells [22, 23]. Moreover, by interacting with the Toll-like receptors (TLRs) and releasing proinflammatory cytokines, such TNF-alpha, IL-1 beta, and IFN-gamma, CD4^**+**^ T-cells are able to activate macrophages, promoting inflammatory reaction [17]. Activated CD4^**+**^ T-cells, isolated from the pancreatic islets of T1DM patients, produce significant amount of interferon-*γ* (IFN-*γ*), contributing to disease progression. Therefore, therapeutic interventions that target CD4^**+**^ T-cells are probably considered beneficial for T1DM patients. T helper cells 1 and 2 (Th1 and Th2) are two other subgroups of CD4^**+**^ T-cells. As Th1 cells produce abundant IFN-*γ* and Th2 cells secrete IL-4 cytokines, they play important roles in promoting autoimmune disease, including T1DM [24]. In addition, clinical data revealed that T1DM patients have increased IL-17-producing cells, which upregulate the level of IL-17 in the circulation as well as Th17 cells that specifically target pancreatic islet *β*-cells [25]. Consistently, another study applied IL-25 and antibodies to IL-17A to NOD mice and showed a reduction in disease progression [26]. Taken together, these studies supported the pathogenic role of Th17 cells in T1DM disease.

### 2.2. Treg Cells

Regulatory T-cells (Tregs) are the critical subset of CD4^**+**^ T-cells, which play roles in both inflammatory and anti-inflammatory environments. Tregs regulate effector cells and diminish the inflammatory response. Several studies have shown that depletion of Tregs favors autoimmunity in mouse autoimmune models. However, during T1DM process, the function of Tregs remains obscure. Waid and Schneider et al. [27, 28] have reported that the number and functions of Tregs in T1DM mouse models and T1DM patients remained normal. In contrast, other researchers have suggested that Tregs from pancreatic lymph nodes of T1DM patients displayed dysfunction when compared to Tregs from peripheral blood [29–31]. Klocperk et al. [32] have investigated a cohort of 38 children with new onset T1DM and found significantly increased number of Tregs. A recent study revealed the beneficial effects of Tregs in inhibiting the progression of T1DM, in which they function to suppress the pathogenic immune cells by lowering the expression of intercellular adhesion molecule-1 (ICAM-1) in the pathologic pancreatic tissue, thus inhibiting the T-cell infiltration [33]. Tregs generate their protective effects by suppressing the synthesis of proinflammatory cytokine IFN-*γ*, which then stimulates self-antigen presentation on pancreatic *β*-cells and induces autoimmunity [33]. Tregs indirectly inhibit the activation and expansion of T-cells and suppress their interaction with DCs during the initial process of T1DM development [34]. On the other hand, Tregs also function to decrease the level of costimulatory molecules present on the surface of DCs, thus limiting the immunogenic activity of DCs [35]. In summary, these results suggest that Tregs regulate the progression of T1DM by affecting multiple steps of T-cell activity, including maturation, expansion, and tissue infiltration.

### 2.3. CD8^+^ T-Cells

CD8^+^ T-cells, also known as cytotoxic T-cells, are an important subtype of T-cells and are enriched in insulitis, mediating the destruction of islet *β*-cells [36–38]. *β*-cells survive only in transplantation recipients, who are immunosuppressed, especially when the CD8^**+**^ T-cells are blocked. In NOD mouse model, both CD4^**+**^ T-cells and CD8^**+**^ T-cells are transferred from donors to induce diabetes [39, 40]. CD8^**+**^ T-cells mediate death of *β*-cells through several signaling pathways. CD8^**+**^ T-cells target pancreatic *β*-cells by recognizing the major histocompatibility complex (MHC) class I molecules and inducing cell death. Cell death cytokines, including IFN-*γ*, are secreted from both CD4^**+**^ T and CD8^**+**^ T-cells, which then trigger the apoptotic cascade through FAS-FASL interaction and lead to the loss of *β*-cells. IFN-*γ* also activates macrophages and produces increasing proinflammatory cytokines, including IL-1b, and TNF [41, 42]. Investigators have suggested the possibility that the significant increase in the preproinsulin-reactive CD8^+^ T-cell level in the pancreatic tissues could destroy the pancreatic insulin-producing *β*-cells [43, 44]. Specifically, the CD8^+^ T-cells target *β*-cell antigens, which are the 65-kilodalton isoforms of glutamic acid decarboxylase (GAD65), insulin, and pancreatic islet-specific glucose-6-phosphatase-related protein (IGRP) that are presented in T1DM patients. Mechanistically, following the increased activity of CD8^+^ T-cells, the level of IL-7 level is increased, which subsequently promotes glucose uptake by elevating glucose transporter 1 (GLUT1) and hexokinase 2 (HK2) levels, deteriorating hyperglycemia B [45]. In addition, CD8^+^ T-cells could secrete membrane-disrupting proteins, such as perforin and granzyme B, which induce *β*-cell apoptosis directly [46]. Therefore, CD8^+^ T-cells are considered as potential subgroup target for T1DM therapy. Tezza et al. [47] have reported that the adenosine triphosphate- (ATP-) gated iron channel P2X7 receptor (P2X7R) is overexpressed on CD8^+^ T-cells in T1DM patients, and these, in turn, function to activate CD8^+^ T-cells upon ATP stimulation. Interestingly, the loss-of-function mutation of the P2X7R prevents the development of T1DM, indicating that the extracellular ATP/P2X7R signaling might play a role in T1DM initiation by supplying energy to CD8^+^ T-cells and may serve as a potential target for T1DM treatment.

### 2.4. iNKT Cells

The invariant NK T (iNKT) cells are a group of T-cells that consists of invariant T-cell receptor (TCR) *α* chain, which recognizes the nonpolymorphic MHC class I-like antigen presenting molecule CD1d [48]. The role of iNKT cells in T1DM remains debatable to date. A previous study showed that increasing number of iNKT cells prevents the development of T1DM. However, Griseri et al. [49] have discovered that high frequency of iNKT cells promote severe insulitis and exacerbate diabetes by enhancing the activity of CD8^+^ T-cells as well as their differentiation into effector cells that produce cytokines, including IFN-*γ*. Driver et al. [50] revealed that iNKT cells play contradictory roles in different genetic mouse models depending on the downstream DC response, whether by inducing immunogenic or tolerogenic events. According to these evidences, targeting iNKT cells to improve the patients' health should be taken into consideration by referring to different genetic background.

### 2.5. B-Cells

*Β*-cells play an essential role in T1DM development in NOD mice. The Ig*μ* null mice that are ablated with *Β*-cells are resistant to T1DM [51], while reconstitution with polyclonal B-cell compartment reverses the resistance of the mice to T1DM development [52]. Consistently, depletion of *Β*-cells by treatment of anti-IgM antibodies prevents disease progression [53]. In contrast, other studies showed that T1DM is developed by transferring the pancreatic islet antigen-specific T-cells into NOD mice without the presence of *Β*-cells [40, 54]. This paradoxical phenomenon is also seen in T1DM patients suffering from Bruton tyrosine kinase (BTK) mutation, which is an X-linked hereditary disorder that causes *Β*-cell deficiency, resulting in agammaglobulinemia (XLA). More importantly, BTK mutation in the particular patient does not lead to complete loss of *Β*-cells [55, 56]. In addition, active B-cells from NOD mice express increased levels of TLR-responsive proteins, which mediate TLR3-induced diabetes protection [57]. Furthermore, clinical trials have identified B-cells as possible therapeutic target for the prevention and reversal of T1DM [58]. In summary, *Β*-cells might participate in the development of T1DM, and depletion of *Β*-cells might be a promising strategy in the treatment of T1DM, although further studies are warranted.

## 3. Innate Immunity in T1D

Adaptive immunity (as discussed above) has always been the focus for scientists in studying the pathogenesis of T1DM. Evidences suggest that innate immunity might also play a role in the development of T1DM. Animal and clinical studies have shown the involvement of innate immune cells, including macrophages, neutrophils, DCs, and NK cells, in T1DM pathogenesis. In this section, their roles in T1DM development are presented.

### 3.1. Macrophages

Considerable studies have shown that macrophage cells are involved in T1DM development both in humans and in NOD mice [59]. Previous study showed infiltration of macrophages into pancreatic islets in NOD mice [60], while blockage of adhesion-promoting receptors on the macrophages inhibited the development of T1DM [60, 61]. Macrophages recruited to the pancreas in T1DM secrete cytokines, including TNF and IL-1*β*, which subsequently mediate apoptosis and destruction of *β*-cells. Macrophages also synthesize and release the cytokine IL-12, facilitating the conversion of naïve T-cells into matured cytotoxic T lymphocytes (CTLs), resulting in T1DM onset [60, 62]. Macrophages have the ability to generate ROS in the pathologic pancreas, leading to cellular apoptosis and destruction and acceleration of T1D [60, 63, 64]. In summary, sufficient evidences have shown that macrophages play an important role in the initial and destructive stages of T1DM development.

### 3.2. Neutrophils

Neutrophils are a kind of short-lived cells of the innate immune system, but play pivotal roles in the initiation and progression of T1D [65]. They have already infiltrated in the pancreas of 2-week-old NOD mice, and the circulating neutrophil count is decreased in T1D patients, indicating that neutrophils may contribute to the earlier initial stage than other immune cell. They have been shown marked abnormalities in both T1D patients and diabetic animal models [66, 67]. Neutrophils can be activated by apoptotic *β*-cells and sustained inflammatory responses by secreting a great deal of proinflammatory cytokines and chemotactic factors [68, 69]. In the pancreatic inflammatory response region, recruited neutrophils can activate macrophages and plasmacytoid DCs and interact with each other to initiate T1D [69, 70]. In fact, the subsets and functions of neutrophils in T1D need to be further investigated, and single-cell sequencing may be a promising method.

### 3.3. NK Cells

NK cells belong to the granular lymphocyte's family and play a role in innate immunity. Distinct from B or T-cells, NK cells have different receptors and specific functions. Due to their ability to kill target cells and interact with antigen-presenting T-cells [71], NK cells are involved in several stages of T1DM development. NK cell-specific genes have significantly distinct expression profile in diabetes-resistant and susceptible mouse model. The number of NK cells that infiltrate the pancreatic islets in T1D as well as the time of their entry acts as indicators of the disease, showing positive correlation with the severity. In addition, scientists were able to slow down the disease onset and progression by depleting NK cells [72]. However, it is still debatable on the exact mechanism as to how NKs participate in T1DM development. One possible explanation for this is the inconsistent role of NKs might be attributed to different NK subsets with different functions, and an imbalance between different subsets might assist in determining the net effect.

### 3.4. DCs

Since 1970s, several researchers have found the presence of APCs in pancreatic islets that underwent transplantation [73], and the survival rate of the recipient mice was increased when these APCs are depleted, suggesting a detrimental effect [74, 75]. Indeed, DCs work at several levels to enhance autoimmunity, promoting T1DM. As potent APCs, DCs capture *β*-cell-derived antigens and present them to lymphocytes that reside in the pancreatic lymph nodes to induce autoimmunity against the pancreatic islet tissue. Dendritic cells secrete high levels of chemokines and cytokines, including IL-15 and IL-12, that trigger an inflammatory cascade, enhance the activity of T-cells, and exacerbate T1DM. In addition, DCs also promote the expression of costimulatory molecules. These data indicated that DCs might be the culprit in the development of T1DM, in which they process self-antigen and present to lymphocytes to facilitate the activity of diabetogenic T-cells while accelerating the inflammatory response. Further studies are required to confirm the role of DCs in T1DM as well as their therapeutic implication [76–78].

### 3.5. Mast Cells

Mast cells make up a very small proportion of innate immune cells, and mainly participate in IgE-mediated allergic diseases. In recent years, many researchers have focused on the regulation of mast cells in autoimmune diseases, especially T1D [79]. Mast cells produce a large amount of the proinflammatory cytokine interleukin-6 (IL-6), which favors differentiation of IL-17-secreting T-cells rather than the tolerogenic Tregs differentiation, contributing to the damage of insulin-producing *β*-cells and the progression of T1D [80, 81]. However, the role of mast cells in the initiation and progression of T1D has been highly controversial. Gutierrez et al. have reported that type 1 diabetes in NOD mice is unaffected by mast cell deficiency, which is quite not consistent with published findings [82]. We cannot draw a comprehensive conclusion with present investigations, and more studies about the functions of mast cells should be conducted in the setting of T1D, especially the related patients.

## 4. Pancreatic *β*-Cells

Pancreatic *β*-cells can produce insulin to sustain the homeostasis of the blood glucose. They are susceptible to microenvironmental factors, such as proinflammatory cytokines, including IL-6, TNF-*α*, and IFN-*γ*, and the attack of autoreactive T-cells in T1D setting [82]. Receptors for the cytokines expressed on *β*-cells are required for the recruitment of macrophages, which, in turn, produce more proinflammatory cytokines, resulting in the death of *β*-cells. In addition, cytokines released by macrophages will interact with T-cells (Tregs) to enhance the autoimmune response and progression of T1D [83]. As a bridge to link innate and adaptive response, *β*-cells can also recognize NK cell, DCs, and other immune cells involved in T1D and interact with them together or respectively. As a kind of nonimmune cells, *β*-cells also have shown very significant immune function similar to antigen-presenting cell (e.g., presenting *β*-cell debris to T-cells in the development of T1D though they have been viewed as a kind of nonimmune cells) [84, 85].

## 5. Immunotherapy in T1DM

Discovered nearly a century ago, insulin has been used as the single definitive treatment for T1DM. However, only 30% of patients receiving insulin treatment have achieved proper blood sugar control, while the remaining are still suffering from hyperglycemia or hypoglycemia and a series of other complications. Therefore, novel methods of immunotherapy probably provide a potential solution to treat the cause instead of the symptom. As a key player in T1DM development, T-cells have always been the focus of research in T1DM treatment [44, 86]. Administration of CD3-specific antibody that targets T-cells to induce T-cell tolerance has significant ability in preventing destruction of *β*-cells in both animal models and humans with recent-onset T1DM [87–89]. Similar treatment outcomes against T1DM have been observed in NOD mice with a combination of antibodies targeting both CD4^**+**^ and CD8^**+**^ T-cells [39]. H6F is an altered peptide ligand that significantly augments CD8^**+**^CD25^**+**^Fop3^**+**^ T-cells in the spleen and pancreas, exhibiting effective inhibition of specific CD8^**+**^ Treg cell response in T1D models [90, 91]. IL-21 is a multifunctional cytokine that is produced by Tfh, Th17, and NK cells and is found to increase in the circulation as well as in the pancreatic tissue in T1DM patients. Anti-IL-21 antibody has been studied in clinical trials in T1DM and other autoimmune patients, and the safety and efficacy of it showed improvement of autoimmune disorders [92]. Combination therapy of anti-IL-21 monoclonal antibody and glucagon-like peptide-1 receptor agonist liraglutide effectively improved blood glucose levels in NOD mice [92]. Granulocyte colony-stimulating factor (G-CSF) arrests DCs in the spleen, resulting in suppression of DCs in NOD mice. These cells promote self-tolerance by imposing effects on CD4^**+**^ CD25^**+**^ Treg cells, as they secrete anti-inflammatory transforming growth factor-*β* (TGF*β*) and suppress the diabetogenic T-cells [59, 93, 94]. IFN*α* is regarded as a crucial compound in both innate and adaptive immunity and played a key role in T1DM development in both clinical patients and laboratory animal models. IFN*α* promotes self-antigen presentation to immune cells and improves recognition of pancreatic *β*-cells by cytotoxic CD8^**+**^ T-cells. Furthermore, the receptor-mediated IFN*α* response induces secretion of chemokines, facilitating the migration of monocytes, T-cells, and NK cells and inducing autoimmunity to the affected tissues [95]. Due to its critical role in the initial step of T1DM development, aiming at IFN*α* and its downstream signaling pathways might be considered as an attractive therapeutic strategy in disease prevention [96]. As mentioned above, receptors presented on *β*-cells are critical for the initiation of T1D; blockage of the key receptors would be a promising therapy. Other researchers have focused on identifying the specific epitopes that are presented by MHC molecules on *β*-cells, which mediated CD8^**+**^ T-cell recognition that resulted in *β*-cell destruction. Such peptidome opens new avenues in developing biomarkers for early disease diagnosis and vaccine for disease prevention [97]. Targeting innate immunity, such as TLR4/MD-2 antibodies, could decrease the adaptive T-cell responses through induction of tolerogenic APCs, indicating that innate immunity might be a potentially preventive and therapeutic target [98]. More effective immunotherapies are still warranted to benefit the T1DM patients.

## 6. Conclusions

**Figure 1:**
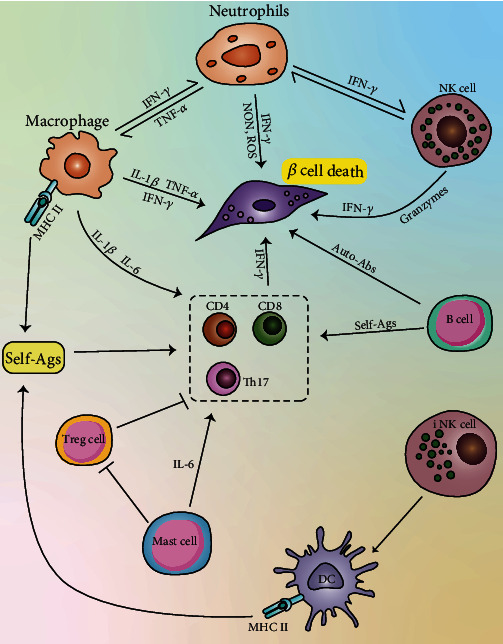
The role of innate and adaptive immunity systems and induction in T1DM patients. The initiation T1D takes place in the pancreas, when dendritic cells (DCs) and macrophages uptake and present *β*-cell antigens to T-cells to activate CD4^+^ and CD8^+^ T-cells. Then, activated CD4^+^ and CD8^+^ T-cells lead to the damage of *β*-cells. At the same time, DCs, macrophages, neutrophils, and NK cells as well as damaged *β*-cells can produce a large number of proinflammatory cytokines such as TNF-*α* and IFN-*γ*, which can directly contribute to *β*-cells' death. And these immune cells interact with each other to enhance their activation state. B-cells present *β*-cell antigens to diabetogenic T-cells and release autoantibodies to damage *β*-cells. iNKT cells can promote the recruitment of DCs. Mast cells facilitate the differentiation of Th17 by producing IL-6, and this effect can be inhibited by Tregs. The crosstalk between innate and adaptive immune cells contributes to the progression or prevention (not shown) of T1D.

Our knowledge on the etiology and pathogenesis of T1DM has been rapidly increasing for over the past few years. Both innate and adaptive immunity plays essential roles in T1DM development (Figure 1), activating and expanding the antigen-recognizing T and B lymphocytes, leading to the ultimate damage of self-insulin producing pancreatic *β*-cells, in which process innate immune cells are also involved. Although immunotherapy shows promising results in restoring self-tolerance and halting destructive autoimmune responses in T1DM, the complex nature of how immune system works and the distinct function of different subtypes of immune cells limited the development of immunotherapy. Studies have focused on the initial process of antibody development and antibody-mediated *β*-cells loss in an effort to recover self-tolerance, while other strategies to preserve *β*-cells are also warranted. In summary, further studies and clinical trials should be conducted to learn more on immune abnormalities in T1DM and then develop more efficient therapeutic strategies to treat T1DM.

## Abbreviations

APC: Antigen-presenting cell
ATP: Adenosine triphosphate
BTK: Bruton tyrosine kinase
ChgA: Chromogranin A
DC: Dendritic cell
GAD65: 65-kilodalton isoform of glutamic acid decarboxylase
GLUT1: Glucose transporter 1
G-CSF: Granulocyte colony-stimulating factor
HK2: Hexokinase 2
IAPP: Islet amyloid polypeptide
ICAM-1: Intercellular adhesion molecule-1
IFN-*γ*: Interferon-*γ*
IGRP: Islet-specific glucose-6-phosphatase catalytic subunit-related protein
IL-1: Interleukin-1
iNKT cell: Invariant natural killer T-cell
MHC: Major histocompatibility complex
NK cells: Natural killer cells
NOD: Nonobese diabetes
ROS: Reactive oxygen species
SCID: Severe combined immunodeficiency
T1DM: Type 1 diabetes mellitus
TGF: Transforming growth factor
Th: T helper
TLR: Toll-like receptor
TNF: Tumor necrosis factor
XLA: X-linked agammaglobulinemia.
